# Research on Cartilage 3D Printing Technology Based on SA-GA-HA

**DOI:** 10.3390/ma16155312

**Published:** 2023-07-28

**Authors:** Yong Chen, Youping Gong, Lijun Shan, Chou Yong Tan, MS Al-Furjan, S. Ramesh, Huipeng Chen, Xiangjuan Bian, Yanda Chen, Yunfeng Liu, Rougang Zhou

**Affiliations:** 1 School of Mechanical Engineering, Hangzhou Dianzi University, Hangzhou 310018, Chinazjhz@163.com (R.Z.); 2 State Key Laboratory of Fluid Power and Mechatronic Systems, School of Mechanical Engineering, Zhejiang University, Hangzhou 310027, China; 3 Department of Mechanical Engineering, Faculty of Engineering, Universiti Malaya, Kuala Lumpur 50603, Malaysia; 4 State Key Laboratory of Mechanics and Control for Aerospace Structures, Nanjing University of Aeronautics and Astronautics, Nanjing 210016, China; 5 Collaborative Innovation Center of High-End Laser Manufacturing Equipment (National “2011 Plan”), Zhejiang University of Technology, Hangzhou 310023, China; 6 Institute of Power Engineering, College of Engineering, Universiti Tenaga National, Km-7, Jalan Ikram-Uniten, Kajang 43009, Selangor, Malaysia; 7 Faculty of Science and Technology, Zhejiang International Studies University, Hangzhou 310030, China; 8 Wenzhou Institute, Hangzhou Dianzi University, 3-4/F, Building B, Zhejiang Yungu, Nanyang Avenue, Yaoxi Street, Longwan District, Hangzhou 325038, China

**Keywords:** 3D bioprinting, additive manufacturing, cartilage regeneration, cartilage tissue engineering scaffold, finite element method

## Abstract

Cartilage damage is difficult to heal and poses a serious problem to human health as it can lead to osteoarthritis. In this work, we explore the application of biological 3D printing to manufacture new cartilage scaffolds to promote cartilage regeneration. The hydrogel made by mixing sodium alginate (SA) and gelatin (GA) has high biocompatibility, but its mechanical properties are poor. The addition of hydroxyapatite (HA) can enhance its mechanical properties. In this paper, the preparation scheme of the SA-GA-HA composite hydrogel cartilage scaffold was explored, the scaffolds prepared with different concentrations were compared, and better formulations were obtained for printing and testing. Mathematical modeling of the printing process of the bracket, simulation analysis of the printing process based on the mathematical model, and adjustment of actual printing parameters based on the results of the simulation were performed. The cartilage scaffold, which was printed using Bioplotter 3D printer, exhibited useful mechanical properties suitable for practical needs. In addition, ATDC-5 cells were seeded on the cartilage scaffolds and the cell survival rate was found to be higher after one week. The findings demonstrated that the fabricated chondrocyte scaffolds had better mechanical properties and biocompatibility, providing a new scaffold strategy for cartilage tissue regeneration.

## 1. Introduction

Cartilage is an essential tissue in animal and humans, and its main components include chondrocytes, cartilage extracellular matrix, and so on. Chondrocytes are metabolically active cells that synthetically transform large amounts of ECM components such as collagen, proteoglycans, as well as hyaluronic acid. Both the internal and external environment in which cartilage resides influences its metabolic activity. Articular cartilage in the human body plays an important role primarily as a support, reducing friction between bones, due to its relatively high frequency of use and susceptibility to trauma as well as inflammation. Healthy cartilage tissue, the new proliferative differentiation, and death of chondrocytes are in a dynamic equilibrium, whereas when the cartilage is in inflammation, injury, or trauma as well as other pathologically induced damages, this equilibrium is broken, eventually leading to the loss of joint. In daily life, osteoarthritis is easily triggered due to cartilage fragility, decreased cartilage function after prolonged exercise, or advancing age. Treatment modalities mainly rely on surgically embedded cartilage scaffolds for repair [1].

Traditional cartilage scaffold materials mainly choose natural biomaterials or artificial materials. Natural biomaterials include sodium alginate, gelatin, chitosan, agar, etc. [2], which are easily accessible and possess a variety of growth factors that can allow chondrocytes to attach to scaffolds and enhance their survival capacity, but the resulting scaffolds have poor mechanical properties [3]. Artificial materials include poly (lactic acid), poly (glycolic acid co lactic acid), polyethylene oxide, hydroxyapatite, etc., the advantages of which can be based on the demand, obtaining scaffolds of different porosity, different mechanical properties of scaffolds, and different degradation rates due to different materials, but the disadvantages are the hydrophobic surface of synthetic polymers, lack of bioactive factors, poor cell adhesion, and acidic metabolites are generated during the degradation process after implantation into humans, which causes a local pH reduction and will cause inflammatory immune responses.

3D printing technology has great advantages in fabricating and maintaining cell scaffolds with complex structures and controlled pore sizes [4,5,6] and combining 3D scanning technology can rapidly fabricate cartilage scaffolds suitable for patients to achieve in situ repair through minimally invasive procedures [7,8]. Hydrogel-based systems have become quite popular in biological 3D printing, and many people have studied the fabrication of chondrocyte scaffolds for cartilage regeneration engineering [9,10]. Systems based on hydrogel have a high water content, are soft like biological tissue, and offer biocompatibility for cell survival [11]. Hydrogels are currently limited in biomaterials that can be utilized in 3D printing and constructing large-scale tissues from hydrogels is not easy due to structural integrity, printability, and mechanical stability [12]. To this end, further studies on novel composite hydrogel formulations, blending natural biomaterials and artificial materials, are needed to fabricate porous cartilage scaffolds with better biocompatibility and mechanical properties to compensate for the defects of single materials. Using bio 3D-printing technology can deposit materials with different physicochemical properties at different places and adjust the concentration of cells as well as growth factors at different places of printing, the degree of adhesion of cells, and the degradation rate of the composite to form cartilage scaffolds rapidly [13]. 

The most widely utilized 3D printing approach for producing tissue engineering scaffolds is extrusion-based 3D printing, due to the fact that a variety of self-configuration materials of the appropriate viscosity can be used [14]. When using extrusion-type 3D printing and printing bio-ink on a hydrogel matrix, the ink must have somewhat discernible viscoelastic behavior under high shear stress in order for the bio-ink to flow through a nozzle under stable circumstances [15]. But bio-ink of hydrogel matrix needs to harden the material by cross-linking to shape the scaffold, during which time the scaffold shrinks and deforms, and both the concentration of cross-linker used, and the cross-linking time affects mechanical properties and shrinkage of the scaffolds [16,17].

After implantation, cartilage scaffolds need to bear mechanical loads in different directions of the human body, so they need strong mechanical properties and toughness. Studies have found that the introduction of hydroxyapatite and other bioactive bio-ceramics into the hydrogel matrix can improve mechanical properties and biological activity. Hydroxyapatite is widely found in animal bones and can participate in metabolism in vivo to promote the repair of defective tissues. However, the disadvantage is that the single hydroxyapatite has poor toughness, is not easy to form, and is easy to block needles. It cannot be used as an engineered cartilage implant [18,19]. In addition to providing a substrate for chondrocyte transport, alginate is also a negatively charged hydrophilic polysaccharide and has been commonly utilized in tissue engineering applications because of its advantages of printability, biocompatibility, low cost, and biodegradability [20,21,22,23,24,25,26]. It has been demonstrated that calcium alginate hydrogels are suitable for transporting adult articular chondrocytes to the sites of cartilage defects in mice [27,28]. CaCl_2_ solution can be used as a crosslinking agent to make alginate brine gels, which have a fast gel rate when in the presence of Ca^2+^ or other bivalent cations [29,30], thus providing an appropriate environment for coating cartilage tissue cells during the layer-to-layer bioprinting procedure [31]. Though hydrogel offers an appropriate environment for cells, it has the significant disadvantage of being less mechanically strong compared to calcified cartilage. Gelatin has the advantages of low manufacturing cost, easy access, easy hydrophilic, good biocompatibility, good surface activity, and biodegradation in the human body [32], mixing with alginate can improve biocompatibility and promote cell growth.

**Figure 1:**
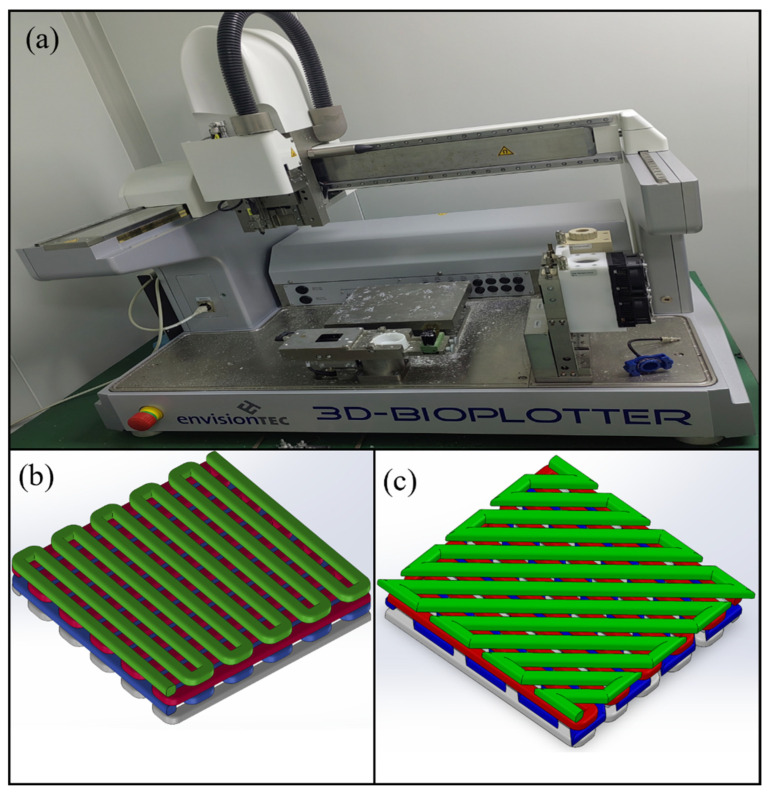
Printing equipment and pretreatment of cartilage scaffold. (**a**) Bioplotter printer; (**b**) 0/90° stacking method; (**c**) 0/45° stacking method.

Therefore, the composite hydrogel of sodium alginate, gelatin, and hydroxyapatite was used to fabricate cartilage scaffolds. The printing equipment uses a 3D-Bioplotter printer manufactured by EnvisionTEC, a German company (Gladbeck, Germany). The preprocessing of the printer and model is shown in Figure 1.

## 2. Materials and Methods

### 2.1. Hydrogel Preparation Process and Material Formula

In this paper, sodium alginate type S100127 (ALADDIN, Shanghai, China), gelatin type BSF180112 (BOSF, Hefei, China), and hydroxyapatite type H139936 (ALADDIN, Shanghai, China) are used. The configuration procedure is as follows:

1. Use a balance to weigh each component of the reagent powder for use;
2. To make it distribute uniformly, slowly add sodium alginate powder to purified water many times at room temperature, then stir continuously to dissolve;
3. Pour the gelatin into cold water, soak it for 10 min, heat it in a water bath at a constant temperature (40 °C), and stir it with a blender. The solution of gelatin and sodium alginate should be mixed and stirred evenly.
4. Add hydroxyapatite powder and stir evenly.

When printing with a 3D-Bioplotter printer, appropriate viscosity of the paste is required to make the printing effect good. Therefore, SA solution, GA solution, and SA-Ga solution of different concentrations should be set for comparison, and the relationship between viscosity and concentration should be analyzed to obtain the solution concentration suitable for the Bioplotter printer. Then, the influence of adding HA material ratio with different content on extrusion lines should be studied to obtain the material ratio suitable for printing.

The viscosity of SA solution and GA solution increases with the increase in concentration. The SA solution with 4%, 5%, 6%, 7%, and 8% concentration was mixed with 2%, 4%, 6%, 8%, and 10% GA solution in the same amount. They were tested with a viscometer, and found the viscosity of the mixture solution of 6% SA and 8% GA to be 9.6, which was within the printable viscosity range of the printer. The printer test found that the mixture solution of 6% SA and 8% GA is suitable for printing. The concentration of SA and GA in the mixed solution is 3% and 4%, respectively.

**Figure 2:**
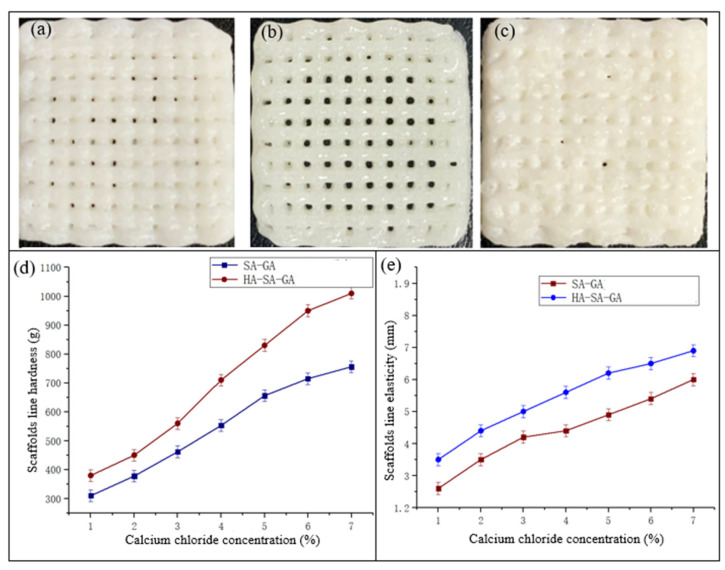
(**a**) 10%HA effect diagram; (**b**) 13%HA effect diagram; (**c**) 16%HA effect diagram; (**d**) hardness comparison diagram of SA-GA and SA-HA-GA support lines; (**e**) comparison of line elasticity of SA-GA and SA-HA-GA supports.

Amounts of 10%, 13%, and 16% HA were added into the prepared SA-GA mixed solution and stirred well. Then, it was put into the barrel to study the lines of the Bioplotter printing extrusion scaffold. Figure 2a–c show the findings. When the HA concentration was 13%, the scaffold was formed evenly and had a high porosity, which was suitable for Bioplotter printing.

The preparation of SA-GA-HA mixed solution and different concentrations of calcium chloride solution was fully cross-linked, as a comparative experiment to analyze the effect of adding HA on the elasticity and hardness of the scaffold lines. From Figure 2d,e, we can notice that the addition of HA significantly increased the hardness and elasticity of the lines of the cartilage scaffold, which was in line with the expected material requirements of the cartilage scaffold, proving the feasibility of the experimental scheme. With the increase in calcium chloride concentration, the hardness and elasticity of the SA-HA-GA composite scaffold line also increased. Overall, the concentration suitable for the crosslinking of support lines in this experimental scheme is 3%. Finally, it was determined that the mixture of slurry contained a sodium alginate concentration of 3%, a gelatin concentration of 4%, a hydroxyapatite concentration of 13%, and a crosslinking agent with a calcium chloride concentration of 3%.

### 2.2. Print Process Model Building

#### 2.2.1. Modeling of Mixed Slurry Fluid Behavior

In order to accurately describe non-Newtonian fluids with yield stresses, the Herschel–Bulkley model is particularly useful. The Herschel–Bulkley model of fluid behavior can be used to simulate the fluid behavior of the slurry mix due to its unique molecular structure and high molar mass of sodium alginate.

(1) $$\tau =K\gamma^{n}.$$

In Formula (1), τ,γ,n are shear stress, shear rate, and behavior index of mixed slurry, respectively. n denotes the degree of non-Newtonian behavior of the fluid. There is a change in shear stress with the shear rate for different types of non-Newtonian fluids. When *n* = 1, a fluid is a Newtonian fluid since its consistency index and viscosity are equal.

#### 2.2.2. Establishment of Needle Extrusion Velocity Model

**Figure 3:**
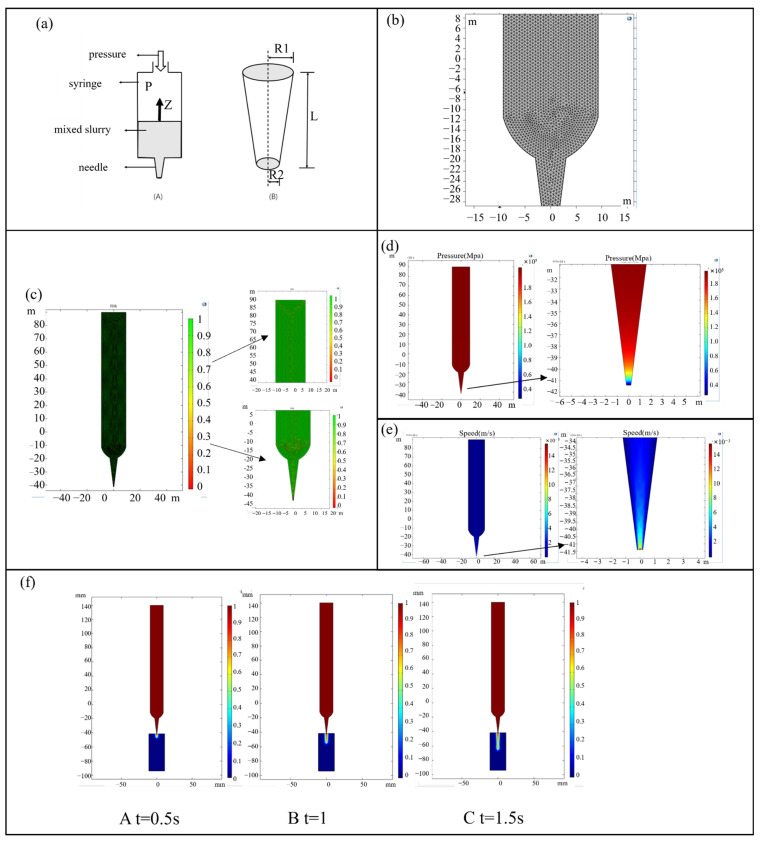
(**a**) Bioplotter print head diagram; (**b**) syringe and syringe grid diagram; (**c**) mesh quality and local mesh magnification diagram; (**d**) simulated pressure gradient diagram and local pressure magnification diagram; (**e**) needle flow rate diagram and local magnification diagram; (**f**) mixing slurry extrusion process diagram from the needle.

As demonstrated in Figure 3a, the Bioplotter printer’s print head can be made simpler, where the configured blend is placed in a needle barrel and is extruded by applying pressure through the top of the needle barrel. Bioplotter air pressure is adjustable from 0 to 5 bar, and the needle outlet diameter is generally selected at 0.4 mm. The printing needle has three different radii: R1, R2, and L, which refers to its vertical length. R1 is the radius of the needle tube, whereas R2 is the radius of the outlet. It is assumed that the flow rate and velocity of mixed slurry in the needle barrel is approximately zero during each extrusion process, since the volume of mixed slurry in the needle barrel is much larger than the volume in the needle. Thus, only the flow rate and flow rate of mixed slurry in the needle barrel are considered when performing the extrusion process. In order to further simplify the model, the experimental scheme made the following assumptions:

1. The mixed slurry flow is completely developed and incompressible.
2. Energy loss and pressure loss in the syringe is ignored.

In the event that the mixed slurry in the syringe is squeezed, the following continuity equation and momentum equation apply:

(2) $$\frac{\partial \rho }{\partial t}+\nabla \cdot \left(\rho u\right)=0,$$

(3) $$\frac{\partial \rho u}{\partial u}+\nabla \left(\rho u⊗u\right)=-\nabla p+\nabla \tau +pg.$$

In Formulas (2) and (3), $\rho =\rho \left(t\right)$ is the density of mixed slurry, $u=u\left(x,y,z,t\right)$ is the velocity vector, $\nabla =\left(\frac{\partial }{\partial x}+\frac{\partial }{\partial y}+\frac{\partial }{\partial z}\right)$ is a partial differential operator, $p=p\left(p,u\right)$ refers to the needle pressure, and τ refers to the stress tensor. In this experiment, it is assumed that the mixed slurry flows are incompressible and stable, and the integral of time is 0. Formulas (2) and (3) can be simplified as follows:

(4) $$\nabla \left(\rho u\right)=0,$$

(5) $$\nabla \left(\rho u⊗u\right)=-\nabla p+\nabla \tau +\rho .$$

The mixed slurry in this experiment is a power-rate non-Newtonian fluid, and the stress tensor is as follows:

(6) $$\tau =K\gamma^{n}=\eta \gamma ,$$

(7) $$\eta =K{\left(\frac{\partial u}{\partial x}\right)}^{n-1}.$$

In Formulas (6) and (7), the apparent viscosity of the fluid is represented by η. By substituting Formulas (4) and (5) into (6), we can obtain:

(8) $$\frac{\partial p}{\partial z}=K\frac{\partial }{\partial x}{\left(\frac{\partial u}{\partial x}\right)}^{n},$$

Formula (8) is integrated twice with *x* to obtain, we can obtain:

(9) $${\left(x\frac{\partial p}{\partial z}-C_{1}\right)}^{\frac{1}{n}}=K^{\frac{1}{n}}(\frac{\partial u}{\partial x}),$$

(10) $$\frac{{\left(x\left(\frac{\partial p}{\partial z}\right)-C_{1}\right)}^{\frac{1}{n+1}}}{\left(\frac{\partial p}{\partial z})(\frac{1}{n+1}\right)}=K^{\frac{1}{n}}u+C_{2}$$

In Formulas (9) and (10), *C*_1_ and *C*_2_ are constants. It depends on the boundary conditions. The maximum velocity of power-rate fluid when flowing through the needle occurs outside the central axis, as follows:

(11) $$\frac{\partial u}{\partial x}=0.$$

Substituting Formula (11) into Formula (9), we can get $C_{1}$ = 0.

(12) $$C_{2}=\frac{(\frac{R_{1}\partial p}{\partial z}{)}^{\frac{1}{n+1}}}{\left(\frac{1}{n+1}\cdot \frac{\partial p}{\partial z}\right)}.$$

Thus, the mixed slurry flow velocity inside the needle is:

(13) $$u=\frac{(x\frac{\partial p}{\partial z}{)}^{\frac{1}{n+1}}-(\frac{R\partial p}{\partial z}{)}^{\frac{1}{n+1}}}{K^{\frac{1}{n}}(\frac{1}{n+1}\cdot \frac{\partial p}{\partial z})}.$$

#### 2.2.3. Extrusion Flow Model of Needle

Compared with the needle, the mixed slurry flow volume in the needle tube is greatly larger than extrusion amount at the needle. When the mixed slurry moves inside the needle, the shear stress received by the slurry on the needle wall is too large, and a slip will occur. Because the slurry has a strong affinity for the wall, the extrusion flow of the needle can be written as:

(14) $$Q=Q_{1}+Q_{2}.$$

In Formula (14), Q is the total extrusion flow of the needle, $Q_{1}$ is the shear flow, and $Q_{2}$ is the slip flow. Assuming that a needle is a tiny unit of radius $R_{2}$, the shear flow at the needle is obtained.

(15) $$Q_{1}=\int_{0}^{R}2\pi uxdx.$$

By substituting the velocity in Formula (14) into Formula (15), the formula of extruded slurry flow rate can be obtained:

(16) $$Q_{1}=\frac{\frac{n+1}{n+2}(\frac{\partial p}{\partial z}{)}^{\frac{1}{n+1}}{R_{2}}^{\frac{n+2}{n+1}}-{R_{2}}^{\frac{n+2}{n+1}\cdot (\frac{\partial p}{\partial z}{)}^{\frac{1}{n+1}}}}{K^{\frac{1}{n}}(\frac{1}{n+1}\cdot \frac{\partial p}{\partial z})}.$$

The slip flow $Q_{2}$ can be obtained by the slip speed and needle area at the needle opening:

(17) $$Q_{2}=u^{\prime \prime }(\pi {R_{1}}^{2}).$$

In Formula (17), the slip flow rate is represented by u′, which can be attained by the above formula:

(18) $$u^{\prime }=\frac{s\tau^{\frac{1}{n}}}{K^{\frac{1}{n}}}.$$

In Formula (18), s is the thickness of slip layer, and the shear force on the mixed slurry is represented by τ. For a tiny round needle unit of length dL, the balance equation of the shear stress of the machine-applied pressure and the slurry mixture can be written as:

(19) $$\pi {R_{1}}^{2}dp=2\pi R_{1}\tau dL.$$

The relationship between shear flow and strain velocity is as follows:

(20) $$\gamma =\frac{4Q_{1}}{\pi {R_{1}}^{3}}.$$

Substituting Formula (20) into Formula (1), the shear stress is:

(21) $$\tau =K(\frac{4Q_{1}}{\pi {R_{1}}^{3}}{)}^{n}.$$

In this experimental scheme, the HA used is nanometer HA, SA particle size is 60 μm, and the thickness of the slip layer is generally 15% of the particle size in the mixed slurry, so the slip layer in this experiment is 9 μm.

The sliding flow rate $Q_{2}$ can be obtained by connecting Formulas (17), (18) and (21).

(22) $$Q_{2}=\frac{sK^{\frac{n-1}{n}}\pi {R_{1}}^{2}4^{n}(\frac{\frac{n+1}{n+2}(\frac{\partial p}{\partial z}{)}^{\frac{1}{n+1}}{R_{2}}^{\frac{n+2}{n+1}}-{R_{2}}^{\frac{n+2}{n+1}\cdot (\frac{\partial p}{\partial z}{)}^{\frac{1}{n+1}}}}{K^{\frac{1}{n}}(\frac{1}{n+1}\cdot \frac{\partial p}{\partial z})}{)}^{n}}{\pi {{R_{1}}^{3}}^{n}}.$$

In combination with Formulas (15) and (21), the extrusion flow *Q* of the needle can be written as:

(23) $$Q=\frac{\frac{n+1}{n+2}(\frac{\partial p}{\partial z}{)}^{\frac{1}{n+1}}{R_{2}}^{\frac{n+2}{n+1}}-{R_{2}}^{\frac{n+2}{n+1}\cdot (\frac{\partial p}{\partial z}{)}^{\frac{1}{n+1}}}}{K^{\frac{1}{n}}(\frac{1}{n+1}\cdot \frac{\partial p}{\partial z})}+\frac{sK^{\frac{n-1}{n}}\pi {R_{1}}^{2}4^{n}(Q_{1}{)}^{n}}{\pi {{R_{1}}^{3}}^{n}}.$$

### 2.3. Simulation Analysis of the Printing Process

By analyzing the fluid characteristics of mixed slurry, mathematical models such as needle extrusion speed, extrusion flow rate, and so on were established, respectively. COMSOL was used to simulate the flow of fluid during the needle extrusion process. Because the flow of mixed slurry in the actual printing process was complicated, the following assumptions were made:

1. Assume that the slurry mix is incompressible and is fully developed inflow;
2. Ignore the influence of gravity on the mixing slurry;
3. Ignore the pressure loss due to the gas transmission of the printing equipment.

For printing with Bioplotter, pressure is subjected to the upper end of the needle tube, assuming that the needle tube is full by composite material, A laminar flow is characterized by pressure inlets and outlet conditions, a non-Newtonian fluid, and pressure inlet and outlet conditions. The fluid simulation, solution, and calculation of the needle extrusion process were carried out by COMSOL 6.0 software, and the mesh was divided into a free triangle mesh. Figure 3b shows the grid division diagram.

COMSOL 6.0 software quantifies the grid quality through 0 and 1. The greener the grid color is, the better the grid quality will be, and the more accurate the simulation results will be. The grid quality diagram is shown in Figure 3c, which proves that the grid quality in this simulation is better.

The process of needle extrusion’s pressure gradient is depicted in Figure 3d. Apply 2 bars of pressure on the needle barrel and set the inlet and outlet of pressure from the syringe to the needle tip. For ease of calculation, assume that the mixture is filled with syringes and needles. The simulated pressure gradient diagram can be seen in Figure 3d, in the actual printing process of Bioplotter, maximum air pressure is at the top of the cylinder. The pressure from the barrel to the needle decreases as the paste is extruded.

Figure 3e represents the flow rate of slurry during extrusion, Figure 3e represents the flow rate of slurry during extrusion, the slurry hardly flows in the barrel and the flow rate is maximum at the tip of the needle. The diagram on the right shows the enlarged flow rate at the tip. It can be seen from the diagram that significantly different velocities occur in the inner wall and center of the needle tip. This is because when the mixed slurry is flowing, the slurry is subjected to a shear force that exceeds a critical value, resulting in wall slip.

Figure 3f shows the extrusion process diagram of mixed slurry in COMSOL using the two-phase flow and horizontal cluster method under the laminar flow module. The inlet boundary is set as the pressure inlet, and the flow is a fully developed flow, in which the mixed slurry is set as fluid 1 and air as fluid 2. During the extrusion process, the swelling phenomenon appeared, and finally, the mixed slurry tended to be stable after a period of extrusion.

### 2.4. Print Parameter Optimization and Bracket Printing

Multiple experiments show that when the temperature is between 20–30 °C, the printed cartilage scaffold line is relatively perfect. When the temperature exceeds 30 °C, the viscosity of the composite solution is too low, the mixed slurry presents swelling and diffusion phenomenon, and the line width of the extruded stent is much larger than the diameter of the needle, which is not conducive to the printing and molding of the cartilage stent. When the temperature is 20–30 °C, the extrusion molding of the scaffold line is good, and the molded scaffold cartilage scaffold has high mechanical properties. Based on multiple experimental analyses, this paper selects the Bioplotter low-temperature printing module to print the cartilage scaffold, and the printing temperature is controlled at 28 °C. Multiple groups of comparison experiments with different needle-moving speeds under different air pressure were set. The needle moving speed was set at 7 mm/s, 9 mm/s, 11 mm/s, 13 mm/s, and 15 mm/s, and the air pressure was 1.2 bar, 1.5 bar, 1.8 bar, and 2.1 bar. The line width of the printed support was counted.

**Figure 4:**
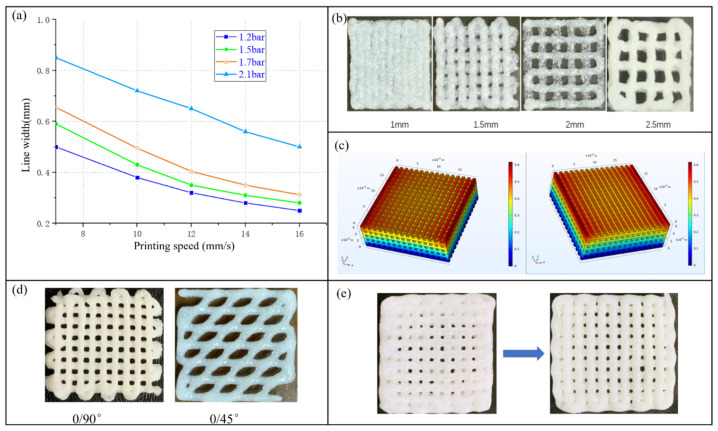
(**a**) Trend diagram of needle-moving speed and support line width under different pressures; (**b**) actual print drawing of different bracket line intervals; (**c**) mechanical stimulation of cartilage scaffold; (**d**) cartilage scaffolds of different internal structures; (**e**) comparison of the stent before and after improvement.

Figure 4a shows the trend diagram of needle-moving speed and support line width at different pressures. As you can see from the figure, the air pressure, needle-moving speed, and support line width are mutually affected. When the air pressure is set unchanged, the faster the needle moving speed, the smaller the support line width. When the needle moving speed is set unchanged, the greater the air pressure, the greater the width of the support line. In the figure, when the needle moving speed is set as a single variable, it can be seen that when the air pressure is from 1.2 bar to 1.7 bar, the width of the support line changes less than that when the air pressure is 2.1 bar. This is because when the pressure is 2.1 bar, the width of the support line changes less. The radial pressure exerted on the line of the support is much greater than the shear force between the mixed slurry and the inner wall of needle. Consequently, when the material is extruded from the needle, the relationship between the needle’s movement speed leads to the accumulation of the material and increases the width of the line of the support. Experiments show that when the needle movement speed is 11 mm/s, suitable line width can be obtained.

Porosity determined the growth of cells on cartilage scaffolds, and appropriate porosity ensured that cells could obtain enough oxygen for proliferation and differentiation. The printing pressure of the printer was set as 1.6 bar, the needle moves at a speed of 11 mm/s, resulting in a 0.32 mm thick cross-section. The influence of the spacing of scaffold lines on porosity was studied. As shown in Figure 4b, due to the existence of objective factors such as gravity, the support line would expand when the mixed slurry was extruded from the needle and deposited on the glass black substrate. The support line was originally round but becoming oval after being deposited on the board. When the scaffold interval was 1 mm, there was basically no hole on the cartilage scaffold because of the diffusion of the mixed slurry, and the porosity was 0. When the scaffold interval was 2 mm, the scaffold had a higher porosity, and when the scaffold line interval was 2.5 mm, the scaffold porosity was also relatively high. The scaffold line was too sparse, leading to the bending of the cartilage scaffold. In conclusion, the reasonable selection of scaffold lines has a crucial impact on the porosity of cartilage scaffolds. In this study, the spacing of scaffold lines was set at 2 mm.

According to the previous analysis, the air pressure and needle moving speed affect the width of scaffold lines, the spacing of scaffold lines affects the porosity, and the width of scaffold lines directly affects the mechanical properties of cartilage scaffolds after molding. Therefore, different stacking modes and spacing of scaffold lines are used to optimize. COMSOL was used to conduct a mechanical simulation of cartilage scaffolds with 0–90° stacking mode and 2 mm separation between scaffold lines. Measure the mechanical properties of the cartilage scaffold by measuring the compression on its surface under a certain pressure. The diameter of articular cartilage in the human body is about 4.82–5.23 cm. In order to facilitate simulation, the diameter of the cartilage is 5 cm. The average body weight in China is 65 Kg, and it is calculated the compression force on the cartilage is 0.324 MPa. In order to simulate the real stress of the cartilage scaffold in the human body, the boundary condition was set as applying pressure to the surface of the cartilage scaffold, the size of which was 0.324 MPa and the direction was vertical. The simulation results are displayed in Figure 4c. It is obvious that when 0.324 MPa pressure is applied above the cartilage scaffold to simulate the force of the scaffold in the body, the cartilage’s deformation is small, indicating that it is theoretically possible to set the printing parameters at the interval of 0/90°–2 mm for the printing of the cartilage scaffold. Cartilage scaffolds with different stacking modes were printed to verify the correctness of the simulation. As shown in Figure 4d, cartilage scaffolds with a stacking mode of 0–90° had the best forming effect.

Another problem exists when Bioplotter is used to print the scaffold. The boundary layer of the cartilage scaffold is easy to accumulate materials. The reason for this problem is that when Bioplotter starts to print, the needle is still moving, and the air pressure device is already supplying air pressure. When many layers are printed, material builds up at the edges of each layer. After the above analysis of this phenomenon, it is proposed to improve the situation by adjusting the extrusion time before printing. After many experiments, parameters are set to lag 1 s before the inlet pressure is printed. The improved printing diagram is shown in Figure 4e, the boundary layer accumulation of bracket lines has been significantly improved.

## 3. Experiments and Results

### 3.1. Test of Mechanical Properties of Cartilage Scaffolds

A universal material testing machine at a working speed of 0.3 mm/min was used to test the mechanical properties of the obtained scaffold. Four groups of stress-strain curves were obtained, elastic modulus and tensile strength were calculated, respectively, as shown in Formulas (24) and (25).

(24) $$E=\frac{\sigma }{\varepsilon },$$

(25) $$\sigma_{bc}=\frac{P}{A}=\frac{P}{\pi R^{2}}$$

**Figure 5:**
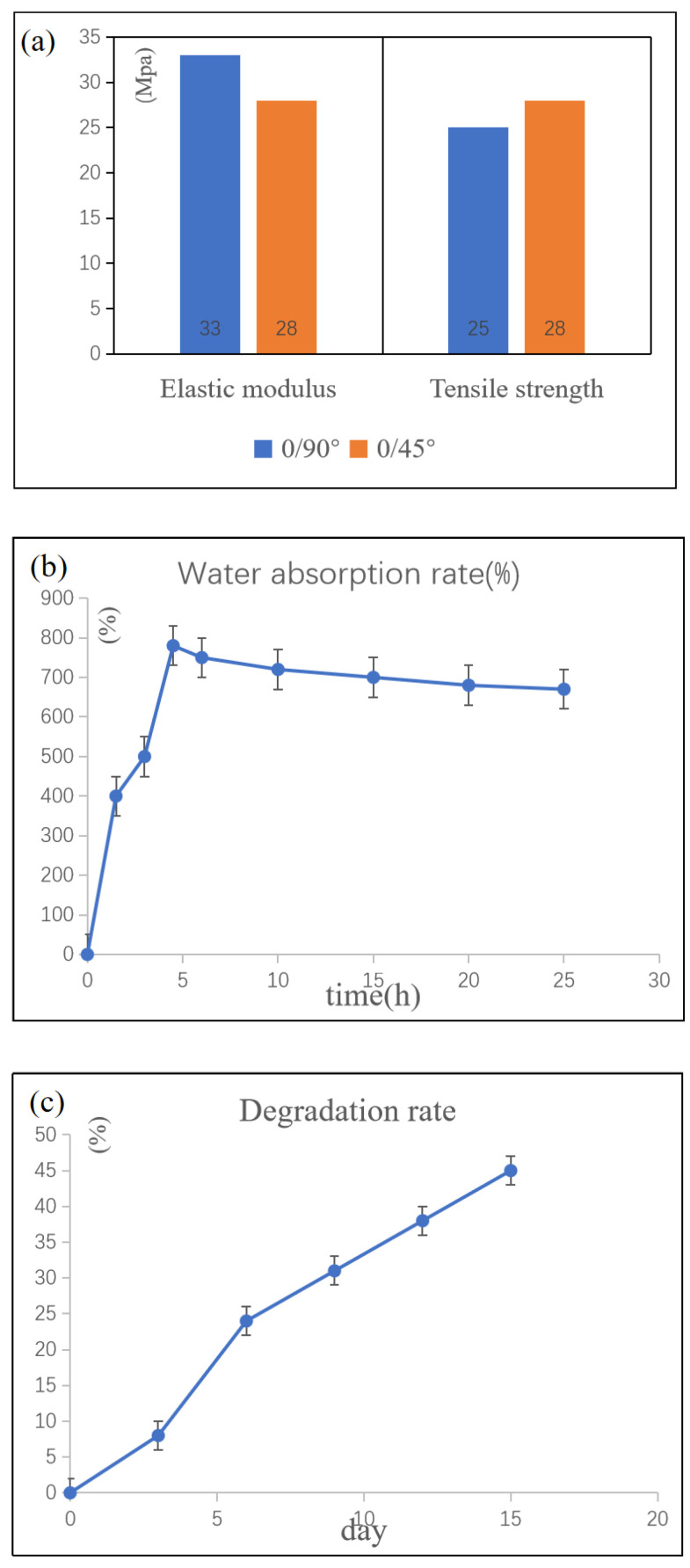
(**a**) Elastic modulus and tensile strength of different stacking mode of cartilage scaffolds; (**b**) water absorption of cartilage scaffolds; (**c**) the relationship between degradability of cartilage scaffolds and time.

In Formula (24), E is elastic modulus, σ is stress, and ε is strain. In Formula (25), $\sigma_{bc}$ is the compressive strength, P is the pressure, and R is the average radius of the sample to be tested. According to the measured data, the relationship between elastic modulus and compressive strength of different stacking types is shown in Figure 5a. For different stacking mode of cartilage scaffolds, 0/90° scaffolds have relatively high elastic modulus and tensile strength. Because the elastic modulus of human knee cartilage in the plumb direction is about 30 MPa, and there is not much need for stretching [33] 0/90° scaffolds are selected to be printed with a 2 mm interval.

### 3.2. Absorbability and Degradability of Cartilage Scaffolds

The printed cartilage scaffold was dehydrated and weighed on an electronic balance with a mass of *m*_1_. The cartilage scaffold was placed in PBS solution to investigate its water absorption rate. Since the temperature of the human body was 37 °C, the temperature of the PBS solution was set at 37 °C. The average water absorption of cartilage scaffolds was measured. The calculation formula of water absorption (*A*) is shown in Formula (26).

(26) $$A=\frac{m_{2}-m_{1}}{m_{1}}.$$

The experimental results in Figure 5b proved that the cartilage scaffold had high water absorption and reached the maximum water absorption rate of 750.92% at 4 h after the cartilage scaffold was immersed in PBS solution. As HA has water absorption, with the increase of soaking time, part of HA dissolves gradually into the pbs solution, the HA content in the gel decreases, and the water absorption decreases.

First, the cartilage scaffold was sterilized by UV irradiation for 1 h, and then the trimethylol aminomethane-hydrochloric acid was configured as a buffer solution with a concentration of 0.05 mol/L, and then the scaffold sample was immersed in the buffer solution at a ratio of 0.03 g/mL. The cartilage scaffold with the mass of *m*_1_ was put into the buffer solution, the temperature was set at 37 °C, the SBF solution was changed every 3 days, and the sample was repeatedly rinsed with ultra-pure water and weighed again after drying treatment, which was recorded as *m*_3_. The weight loss rate (*W*) was calculated as Formula (27).

(27) $$W=\frac{m_{3}-m_{0}}{m_{0}}\times 100\%.$$

From Figure 5c, it can be concluded that the degradation rate of the scaffold does not change much as the number of days increases. Such uniformity of the SA-GA-HA composite cartilage scaffold in this study is a new material scheme. The feasibility of this experimental scheme was tested by its degradability, which was tested by investigating the changing in weight of scaffold in the buffer solution. During the degradation of the scaffold, stable mechanical properties of the cartilage scaffold could be guaranteed. The degradation rate showed a small improvement on the 4th to 6th day. This was because, after three days of degradation, nano HA particles in the cartilage scaffold were decomposed into the solution faster than SA and GA, so the cartilage scaffold showed a faster degradation rate.

### 3.3. In Vitro Cell Assay

**Figure 6:**
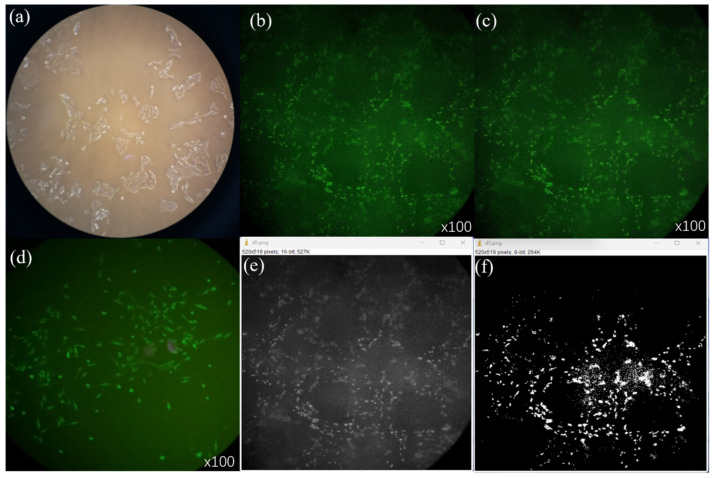
Cell fluorescence. (**a**) are cells in the medium under a microscope; (**b**,**c**) are fluorescence images of scaffold cells in different fields under a 100× microscope; (**d**) are fluorescence images of individual cells under a 100× microscope; (**e**,**f**) is the image processed by ImageJ software.

The scaffolds were soaked in 75% alcohol for 1 h for disinfected and taken out and placed in a vacuum dryer for 24 h, then washed three times in PBS. A total of 1 mL culture solution containing ATDC5 cells (1 × 105 cells/mL, Shenggong Biotechnology Co., Ltd., Shanghaia, China) was dripped onto the scaffold, and 1 mL culture solution (without cells) was added to each pore plate. The inoculated scaffold was transferred to the incubator (37 °C, 5% CO_2_), and allowed cells to freely adhere to the scaffold and grow freely in the next 3 days. A vacuum pump was used to remove the culture liquid, and PBS was used to wash the support again. The same vacuum pump was used to remove the residual waste liquid. Each well was filled with 250 μL Calcein AM assay solution and transferred to an incubator (37 °C, 5% CO_2_) for 30 min under light protection. The scaffold was removed, and the fluorescence was detected with a fluorescent enzyme label instrument. The staining effect was observed under the microscope and the images were taken, as shown in Figure 6b.

Fluorescence images showed that the cells had a higher survival rate and were evenly distributed around the scaffold, indicating that the scaffold was more suitable for chondrocyte growth and that the scaffold cytotoxicity was lower. The ImageJ software was applied to count the cells. First, the image was converted into a 16-bit format to obtain the grayscale map as shown in Figure 6e. The Threshold command was used to adjust the threshold value, remove the background, select the nucleus, and fill the gaps in the nucleus with the fill holes command. Interrupt the overlap of the nuclei with the watershed command and the resulting image is shown in Figure 6f, then the analyze–analyze particles command is used to count automatically. Although the results of cell counting using ImageJ software were greatly affected by the brightness of the images, the results were still of reference significance.

**Figure 7:**
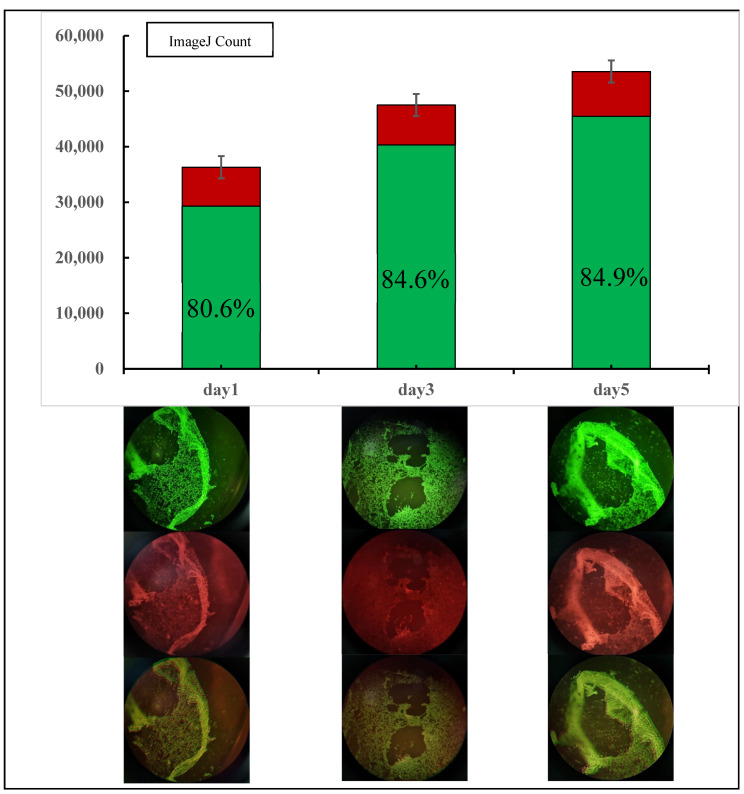
Group experiment results. Green represents living cells, while red represents dead cells.

Three control groups were set up, and the same batch of resuscitated cells was cultured for 1 day, 3 days, and 5 days, respectively. Calcein-AM and PI mixed working solution was added to them for cell staining (Calcein-AM and PI were provided by Yisheng Biotechnology (Shanghai) Co., Ltd., Shanghai, China). Using ImageJ software, dead and alive cell technology was used to obtain Figure 7. In the bar chart. As can be seen from the column, the number of cells and their survival rate increased with the increase in culture time. However, since the cells used were adherent cells, the growth rate was fast, and the space of the 24-hole plate used was small, it was almost overgrown by the fifth day, and most of the dead cells died because of insufficient growth space. Due to the expansion and deformation of the scaffold after adding the staining solution, there was no obvious scaffold structure under the microscope.

## 4. Conclusions

In this paper, SA, GA, and HA three materials were used to study the ratio of cartilage scaffold materials and manufacturing technology, including the establishing a mathematical model of extrusion process, the analysis of the fluid type after the mixing of the three materials, the study and optimization of the printing process parameters, and the mechanical properties of the prepared scaffold model and in vitro biological experiments. The mechanical performance test shows that the fabricated cartilage scaffolds have high elastic modulus and tensile strength, which can meet the actual demand. The results of in vitro cell tests showed that the formulation and fabrication process of the scaffold were not cytotoxic, and the scaffold had good biocompatibility, which was conducive to the adhesion and growth of chondrocytes. In conclusion, this study provides a new strategy for cartilage tissue regeneration by designing a new hydrogel formula and using a 3D printing process to form scaffolds.

## Data Availability

Data available on request due to restrictions eg privacy or ethical. The data presented in this study are available on request from the corresponding author.
